# Metagenomic Sequencing Elucidated the Microbial Diversity of Rearing Water Environments for Sichuan Taimen (*Hucho bleekeri*)

**DOI:** 10.3390/genes15101314

**Published:** 2024-10-12

**Authors:** Qinyao Wei, Zhaobin Song, Yeyu Chen, Huanchao Yang, Yanling Chen, Zhao Liu, Yi Yu, Quanyu Tu, Jun Du, Hua Li

**Affiliations:** 1 Fisheries Research Institute, Sichuan Academy of Agricultural Sciences, Chengdu 611730, China; weiqinyao@scsaas.cn (Q.W.); cyyleaf@126.com (Y.C.); yhc123@scsaas.cn (H.Y.); cqpcy123@scsaas.cn (Y.C.); lz754299716@163.com (Z.L.); 17784411664@163.com (Y.Y.); tuquanyu-12@163.com (Q.T.); dujun9100@126.com (J.D.); 2 Key Laboratory of Bio-Resources and Eco-Environment of Ministry of Education, College of Life Sciences, Sichuan University, Chengdu 610065, China; zbsong@scu.edu.cn

**Keywords:** *Hucho bleekeri*, metagenomic sequencing, water environment, microorganism

## Abstract

Background: Sichuan taimen (*Hucho bleekeri*) is a fish species endemic to China’s upper Yangtze River drainage and has significant value as an aquatic resource. It was listed as a first-class state-protected wild animal by the Chinese government due to its very limited distribution and wild population at present. Methods: To elucidate the diversity of microorganisms in rearing water environments for *H. bleekeri*, metagenomic sequencing was applied to water samples from the Maerkang and Jiguanshan fish farms, where *H. bleekeri* were reared. Results: The results revealed that Pseudomonadota was the dominant phylum in the microbial communities of the water samples. Among the shared bacterial groups, Cyanobacteriota, Actinomycetota, Planctomycetota, Nitrospirota, and Verrucomicrobiota were significantly enriched in the water environment of Jiguanshan (*p* < 0.01), while Bacteroidota was more enriched in that of Maerkang (*p* < 0.01). Additionally, the Shannon diversity and Simpson index of the microbial community in the water environment of Maerkang were lower than in that of Jiguanshan. Conclusions: The present study demonstrated the similarities and differences in the microbial compositions of rearing water environments for *H. bleekeri*, which are expected to benefit the artificial breeding of *H. bleekeri* in the future.

## 1. Introduction

The Sichuan taimen (*Hucho bleekeri*) is a critically endangered fish endemic to the Yangtze River drainage in China and is listed on the Red List of Threatened Species compiled by the International Union for Conservation of Nature (IUCN) [1]. It is characterized by a large body size and predatory behavior and thus occupies the top of the aquatic food chain [2]. Its late sexual maturation, and human activities have brought this species to the brink of extinction [3,4]. Successful ultrasonography-assisted artificial reproduction of *H. bleekeri* has great significance for species preservation [5].

The quality of aquaculture water is critical for the health of aquatic species, as microorganisms play key roles in nutrient cycling and ecosystem function [6,7]. Maintaining the number and variety of microorganisms in the aquatic environment not only plays an important role for fishes living in aquaculture ecosystems but also may have an impact on public health [8]. The presence of *Aeromonas* and Pseudomonadaceae in a particular abundance within an aquaculture water environment has been observed to promote the growth of crustaceans. However, it is noteworthy that certain species of *Aeromonas*, *Acinetobacter*, *Arcobacter*, *Flavobacterium*, *Clostridium*, and *Vibrio*, among others, have the potential to cause disease in crustaceans when present in significant numbers [7,9]. Water microorganisms have been shown to influence primarily the skin community of salmonids, which is further exemplified during their migration from freshwater to saltwater [10]. On the other hand, some aquatic bacterial that have the potential to cause disease in fishes, humans, and other species—such as *Coxiella burnetii*, *Flavobacterium columnare*, *Legionella birminghamensis*, and *L. pneumophila*—originate from ornamental fish tanks [11].

Previously, conventional culture methods and physiological and biochemical characteristics were utilized for the characterization of microorganisms in fish. Through these techniques, Montes and colleagues identified 53 strains of facultative anaerobes, belonging to the genera *Vibrio*, *Aeromonas*, and *Listonella*, within the skin and aqueous environment of *Scophthalmus maximus* [12]. Nevertheless, conventional culture techniques necessitate a considerable investment of time and personnel for the isolation and identification of bacteria, and they fail to fully capture their diversity even when diverse media are employed.

In recent years, metagenomic sequencing has emerged as a significant area of research in microbiology, expanding the scope of investigation for scientists. This technology has been widely employed in microbial ecology, encompassing microbial diversity exploration, metabolic analysis, ecological function research, environmental monitoring, health assessment, and the discovery of new genes and disease research, among other applications. Sáenz et al. [13] discovered through metagenomic sequencing that oral antibiotics increased the prevalence of antibiotic resistance genes (ARGs) and motile genetic factors (MGFs) in the gut microorganisms of *Piaractus mesopotamicus*. This finding suggests that the transfer of ARGs via MGFs may contribute to the dissemination of ARGs in aquaculture. Riiser et al. [14] demonstrated that the ecological niche is a pivotal factor influencing the intestinal microbial composition of fish within the family Gadidae. Moreover, Bharti et al. [15] employed metagenomic techniques to investigate the impact of the polluted Yamuna River on the gut microbial communities of two invasive fish species. In conclusion, metagenomic sequencing represents a powerful tool for advancing our understanding of the intricate relationships between microorganisms and their environment, as well as their role in health and other biological processes.

This study aimed to gain insight into the microbial composition and variations in different water environments for *H. bleekeri* by using the Illumina HiSeq platform for metagenomic sequencing and to identify the dominant and beneficial microbial communities that could aid the survival of *H. bleekeri* in artificial environments.

## 2. Methods and Materials

### 2.1. Sample Collection

*Hucho bleekeri* were reared in two isolated fish farms, namely, Maerkang (32°13′ N, 101°36′ E) and Jiguanshan (30°47′ N, 103°23′ E). Detailed context regarding the water environmental conditions of these two fish farms is provided in Supplementary Table S1.

A total of eighteen water samples were obtained, of which nine were collected from Maerkang on 15 April 2024 and the other nine were obtained from the Jiguanshan on 26 April 2024. All water samples were collected from the ponds for adult fish. Water samples were filtered using a 0.45 μm filter unit (Joanlab, Huzhou, China). Each filter cartridge is designed to collect microorganisms from a single water sample collection container, with a capacity of 1.5 L. Upon completion of the filtration process, the filter cartridge was transferred directly to a 15 mL centrifuge tube and stored at −80 °C until further processing was initiated.

### 2.2. DNA Extraction, Quantification, and Metagenomic Sequencing

Metagenomic DNA was extracted from the water samples using Tiangen DNA Extraction Kit (Beijing, China) according to the methodology set forth by the manufacturer. Once the DNA concentration test was completed, the library was constructed and the subsequent metagenomic sequencing was implemented using Illumina technology.

### 2.3. Data Preprocessing and Metagenome Assembly

Fastp software (version 0.23.1) was utilized for preprocessing the raw data generated by the Illumina sequencing platform. Paired reads were discarded when either read contained adapter contamination, more than 10 percent uncertain nucleotides, or more than 50 percent low-quality nucleotides (base quality less than 5).

In light of the possibility of host contamination in samples, it was essential to perform BLAST searches of the clean data against the host database to filter out any reads that may have originated from the host. Bowtie 2 software (version 2.2.4) was used to eliminate host contamination in the samples, with the following parameters: end-to-end, -sensitive, -I 200, and -X 400 [16,17]. MEGAHIT software (version 1.2.9) was subsequently used for analyzing the assembly data [18].

### 2.4. Functional Prediction

MateGeneMark (version 2.1) was employed to predict the functional repertoire of bacterial communities in water samples [16]. The Kyoto Encyclopedia of Genes and Genomes (KEGG) (version 2.1.6) database was used for gene functional prediction.

### 2.5. Statistical Analysis

To determine the variation in alpha diversity within the water samples, the ACE, Chao1, Shannon, Simpson, observed species, and good coverage indices were calculated and visualized using R software (version 2.15.3). To demonstrate differences in the alpha diversity indices between samples, a histogram was generated to reflect the median, dispersion, and maximum and minimum values of species diversity for each sample. Simultaneously, the Kruskal–Wallis test was used to determine whether differences in species diversity between samples were significant (*p* < 0.05).

Beta diversity analysis was used to assess differences in the structure of microbial communities and to compare species composition and diversity in different water environments, further revealing ecosystem function and stability. The Bray–Curtis distance metric was used for beta diversity analysis [19]. Principal component analysis (PCA), principal coordinate analysis (PCoA), and nonmetric multidimensional scaling (NMDS) were employed to visualize changes in the microbial community structure between the samples. PCA, PCoA, and NMDS dimensionality reduction maps were generated using R software (version 2.15.3). ANOSIM is a non-parametric test used to assess whether the differences between groups are significantly greater than those within groups, with an R-value close to 1 indicating a notable disparity in similarity (high within-group and low between-group similarity), and a permutation test is performed at each taxonomic level to obtain a *p*-value. MetaGenomeSeq and linear discriminant analysis effect size (LEfSe) analysis were used to search for species differences between samples. MetaGenomeSeq analysis was used to perform a permutation test between samples at each taxonomic level and obtain a *p*-value. LEfSe software (version 2.15.3) was used for LEfSe analysis (LDA score of 4 by default) [20].

## 3. Results

### 3.1. Microbial Composition in the Two Water Environments

Following quality control filtering and the removal of primers, chimeras, and singletons, a total of 221.45 G sequence reads were obtained from 18 water samples, with an average of 12.30 G per sample and a range of 11.91 G to 12.89 G (Supplementary Table S2).

**Figure 1:**
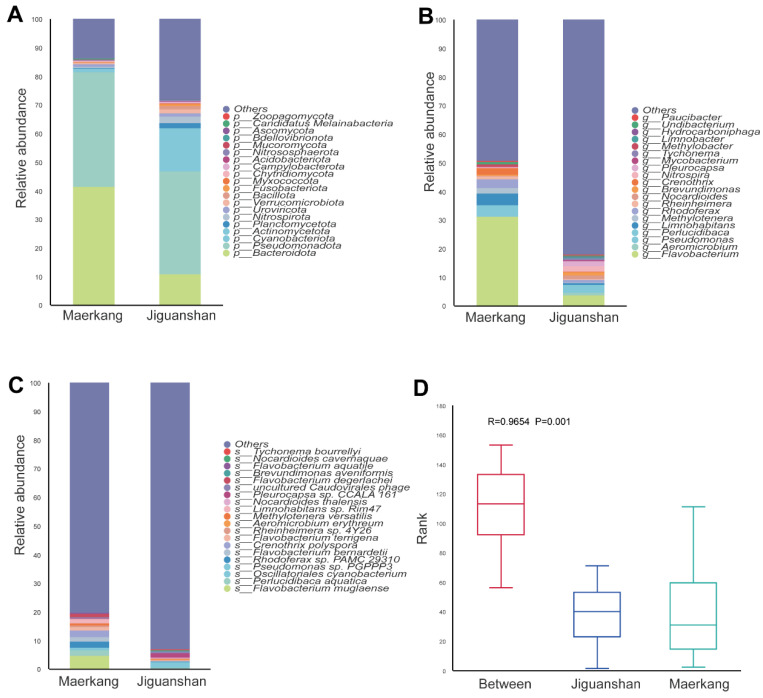
Histogram of the relative abundance of the top 20 species at the phylum level (**A**), genus level (**B**), and species level (**C**). (**D**) ANOSIM between water samples from the two fish farms.

The results of metagenomic sequencing revealed that the dominant bacterial groups in the water samples from the Maerkang and Jiguanshan fish farms were Bacteroidota and Pseudomonadota. The relative abundance of Bacteroidota accounted for 41.37% in Maerkang and 10.77% in Jiguanshan, and that of Pseudomonadota was 39.99% in Maerkang and 36.10% in Jiguanshan, respectively (Figure 1A and Supplementary Table S3). Moreover, the relative abundance of Cyanobacteriota in Jiguanshan (10.55%) was greater than in Maerkang (0.67%). The relative abundances of Actinomycetota, Planctomycetota, and Nitrospirota were greater in Jiguanshan (4.48%, 1.66%, and 2.29%, respectively) than in Maerkang (0.65%, 0.29%, and 0.41%, respectively) (Figure 1A and Supplementary Table S3).

At the genus level, the microbial compositions in Maerkang and Jiguanshan were different (Figure 1B). The top five genera in terms of relative abundance in Maerkang were *Flavobacterium* (31.20%), *Limnohabitans* (4.08%), *Rhodoferax* (3.06%), *Crenothrix* (2.31%), and *Pseudomonas* (2.01%), respectively (Figure 1B and Supplementary Table S5). In Jiguanshan, the top five genera were *Flavobacterium* (3.74%), *Pseudomonas* (2.01%), *Nitrospira* (1.94%), *Pleurocapsa* (1.82%), *Nocardioides* (1.20%), and *Brevundimonas* (1.18%), respectively (Figure 1B and Supplementary Table S5).

At the species level, the microbiome differed between the two environments (Figure 1C). In Maerkang, the dominant bacteria were, in order of prevalence, *Flavobacterium muglaense* (4.64%), *Crenothrix polyspora* (2.26%), and *Rhodoferax* sp. *PAMC 29310* (2.18%) (Figure 1C and Supplementary Table S6), while in Jiguanshan, the dominant bacterial flora species were *Pleurocapsa* sp. *CCALA 161* (1.60%), *Oscillatoriales cyanobacterium* (1.34%), and *Pseudomonas* sp. *PGPPP3* (0.72%) (Figure 1C and Supplementary Table S6). ANOSIM revealed significant discrepancies between the water samples obtained from the two fish farms (Figure 1D, R = 0.9654, *p* < 0.01).

### 3.2. Microbial Diversity in the Two Water Environments

**Figure 2:**
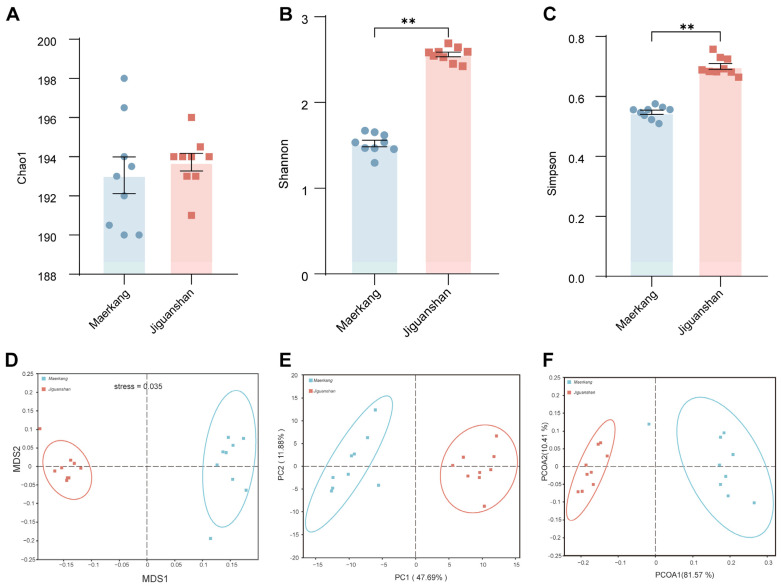
Comparison of the alpha diversity of microbiomes across the samples based on the Chao1 (**A**), Shannon (**B**), and Simpson (**C**) indices. NMDS (**D**), PCA (**E**), and PCoA (**F**) show the microbial community differences between the two water environments. ** *p* < 0.01.

The values of Good’s coverage estimator for all samples were equal to one, indicating that sequencing coverage was successfully achieved and that the species identified were representative of the sampled population. Significant differences were observed in the alpha diversity of the microorganisms between water samples collected from the Maerkang and Jiguanshan fish farms (Figure 2A, *p* > 0.05; Figure 2B,C, *p* < 0.01, Supplementary Table S7). The Shannon and Simpson indices of water samples from Jiguanshan were significantly greater than those of samples from Maerkang (Figure 2A, *p* > 0.05; Figure 2B,C, *p* < 0.01, Supplementary Table S7).

NMDS demonstrated that the samples from Maerkang and Jiguanshan were separately clustered (Figure 2D). Furthermore, the PCA of the microbial community indicated that the water samples from the two farms exhibited relatively distant clustering (Figure 2E). The results of the PCoA were analogous to those of the PCA, with no intersection between the water samples from the two regions (Figure 2F).

### 3.3. Microbial Differences in the Two Water Environments

**Figure 3:**
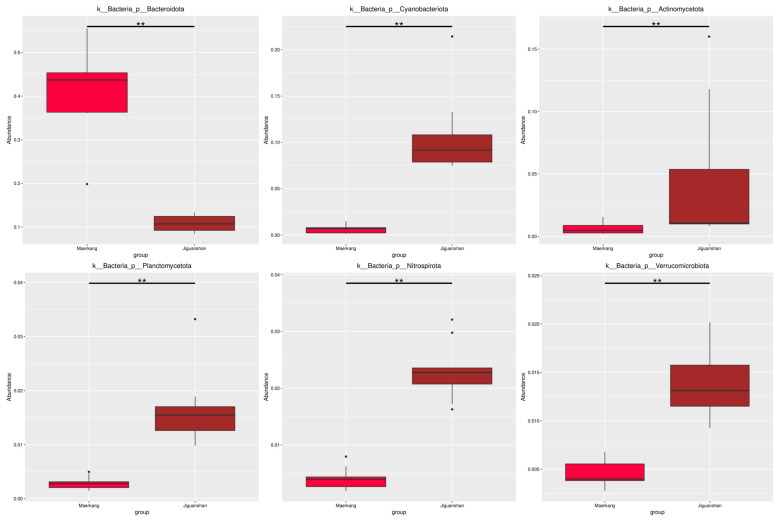
Abundances of microbes at the phylum level (top six) in two different water environments at two fish farms based on MetaGenomeSeq analysis. ** *p* < 0.01.

The results of the MetaGenomeSeq analysis revealed that the abundance of Bacteroidota in Maerkang was significantly greater than that in Jiguanshan at the phylum level (top six) (Figure 3, *p* < 0.01). However, Cyanobacteriota, Actinomycetota, Planctomycetota, Nitrospirota, and Verrucomicrobiota were more abundant in Jiguanshan (Figure 3, *p* < 0.01).

### 3.4. Microbial Function Composition in the Water Environment

**Figure 4:**
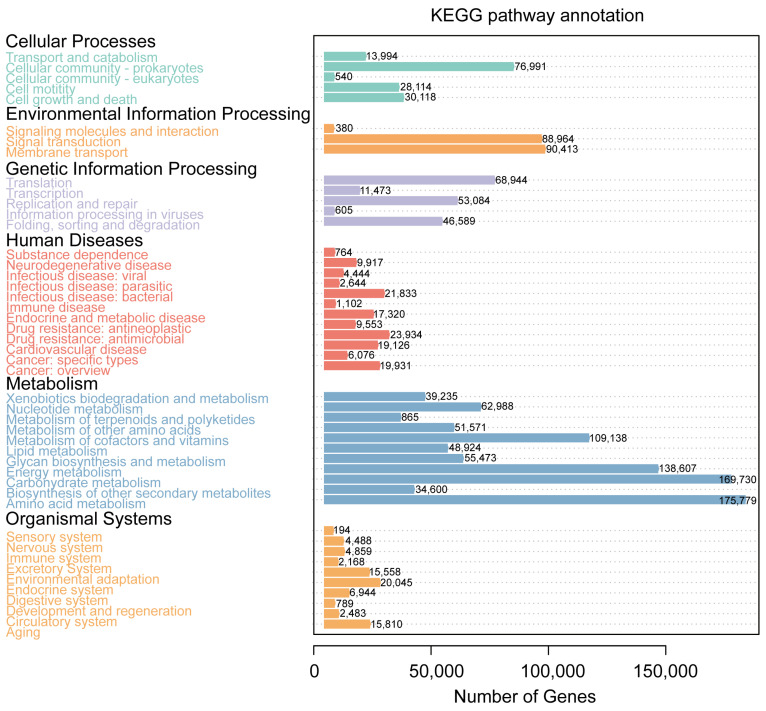
The KEGG functional enrichment analysis results of the microbiome in water samples.

A comparison with the KEGG database revealed that the microorganisms in the water samples were predominantly enriched in pathways associated with metabolism, genetic information processing, environmental information processing, cellular processes, human diseases, and organismal systems (Figure 4). A further comparative analysis at KEGG level 2 revealed that the five most abundant metabolic pathways were, in descending order, amino acid metabolism, carbohydrate metabolism, energy metabolism, metabolism of cofactors and vitamins, and membrane transport (Figure 4).

### 3.5. Microbial Diversity in Water Environments at Different Age Stage

We performed LEfSe analyses to identify the bacterial taxa (at all possible taxonomic levels) that were significantly associated with each water sample, which determined the differences in levels over different years. LEfSe analysis results revealed that the most significant discrepancies in the phylogenetic structure of the microbial communities across the different years were observed at the order and family levels.

**Figure 5:**
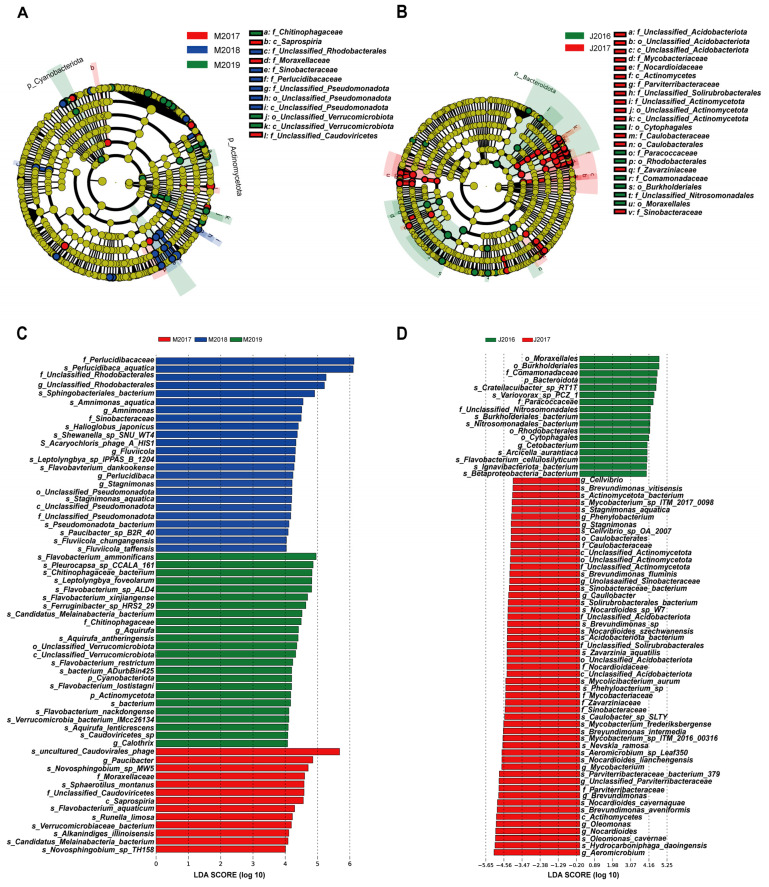
(**A**) LEfSe analyses showed that the microbial differences between water samples from Maerkang in different breeding years from 2017 to 2019 were greater at the family and class levels. (**B**) LEfSe analyses showed that the microbial differences between water samples from Jiguanshan in different breeding years from 2016 and 2017 were more at the family and order levels. (**C**,**D**) The bar chart generated by metagenomic sequencing highlights the significant differences in microorganisms present in water samples from different years at the Maerkang and Jiguanshan fish farms. LDA score equals 4.

The results of the LEfSe analysis revealed that each group exhibited a distinct microbial community structure in their water samples. The evolutionary branch diagram of all taxa is presented in Figure 5A,B, which illustrates the diversity of microbial communities in water samples from the different fish farms. A total of 61 taxa (encompassing five grading levels) were identified as being shared among the M2017, M2018 and M2019 groups in Maerkang (Figure 5C). At the class level, Saprospiria was more abundant in the M2017 group than in either the M2018 group or the M2019 group. In Jiguanshan, 69 taxa (comprising five grading levels) were shared between the J2016 and J2017 groups (Figure 5D). The relative abundances of Mycobacteriaceae, Nocardioidaceae, Parviterribacteraceae, Caulobacteraceae, Zavarziniaceae, and Sinobacteraceae in the J2017 group were greater than those in the J2016 group at the family level.

## 4. Discussion

Fish live in water from hatching, and their gut and skin microorganisms are closely related to their living environment. The microbiotas in the aquatic living environment of fish are significantly distinct from and more diverse than those on the skin and gills of many fish species [21]. Many researches have demonstrated that bacterial diversity in aquatic living environments is greater than that on the skin [22,23,24], the gills [24,25], the stomach [24], and the gut [24,26,27,28] and throughout the larval stage [29] of fish. Furthermore, the spatial patterns of bacterial community composition frequently exhibit a distance–decay relationship, whereby the dissimilarity of communities increases with geographic distance [30]. In this study, DNA samples were extracted directly from two water environments housing *H. bleekeri*.

The results demonstrated that *Pseudomonadota* was one of the dominant bacterial groups in both groups of water samples, which was consistent with the findings of the bacterial community structure of tilapia and grouper breeding ponds reported by Gilbert et al. [31], Zhang et al. [32], and Xiong et al. [33]. Stevens et al. [34] reported that β-ascomycetes are typically the dominant flora in lakes and rivers, whereas α- and γ-ascomycetes are more abundant in seawater. The results of the present study revealed that β-ascomycetes constituted a relatively high proportion of the microbial community in two water environments, which corroborates the hypothesis proposed by Stevens et al. [34]. β-Ascomycetes release important nutrients such as nitrogen and phosphorus through the decomposition of organic matter and mineralization, thereby promoting the cycling and supply of basic nutrients in lakes and rivers. This plays an important supporting role for other organisms in aquatic ecosystems such as algae and planktonic animals [34,35]. In contrast, *α-* and γ-ascomycetes are more prevalent in marine environments, potentially due to their capacity to adapt to higher salinity conditions and more efficiently utilize nutrients in seawater [34].

Water samples from Maerkang were dominated by Bacteroidota, whereas those from Jiguanshan were not. Bacteroidota represents a significant microbial group within aquatic ecosystems, serving as a primary consumer of dissolved organic matter [36]. In an aquaculture water environment, the deposition of residual food and feces that do not decompose quickly may increase the level of bacteriophage anomalies [37]. The prevalence of flora feeding on degradable organic matter, such as Flavobacteria, is crucial for the expeditious decomposition of organic matter in aquatic ecosystems and the alleviation of stress in aquatic environments [38]. In this study, the most prevalent phylum in Maerkang was Flavobacterium, which may be attributed to the high farming density, resulting in an increased concentration of fecal matter in the water environment. The increase in the amount of feces leads to an increase in the number of Flavobacteria.

The abundance of *Cyanobacteriota*, a widely distributed bacterial genus found in aquatic environments, is closely associated with the eutrophication of the water environment, which is caused by the input of nutrients [39]. *Actinomycetota* represents a significant component of the freshwater bacterioplankton community [40]. The present study revealed that Cyanobacteriota and Actinomycetota were highly abundant in Jiguanshan, which may be attributed to the presence of households situated upstream of Jiguanshan’s water source. The daily wastewater of residents is conveyed downstream by the prevailing water flow. These daily water sources typically exhibit high nitrogen and phosphorus concentrations, which can precipitate eutrophication of the water environment. However, the area upstream of Maerkang’s water source is devoid of human settlements.

Studies have reported differences in the composition of embryonic and gut microbial communities during the growth phase of Atlantic salmon (*Salmo salar*) [41]. Similar results have been reported in gill, water, and gut samples from other species, including reef fishes [25] and *Danio rerio* [42]. In the present study, although the water samples were collected at closely spaced timepoints, the microorganisms in the water samples from each farm also differed between timepoints. These findings suggest that the microbial community composition of the water samples was highly dynamic and varied with age.

In aquaculture, microbial diversity is closely related to the stability of the aquatic environment. A reduction in microbial diversity within a water environment increases the risk of aquaculture disease outbreaks [43]. In this study, the Shannon and Simpson indices, which are used to assess the diversity of bacterial communities in water samples, were significantly lower in Maerkang than in Jiguanshan. This study demonstrated that the microbial diversity in Maerkang was inferior to that in Jiguanshan. The water samples from Jiguanshan had a more diverse microbial population, which may be because Jiguanshan is an open area with greater exposure to sunlight and increased bacterial production during phytoplankton blooms, resulting in greater exposure of chloroplasts to light, which increases primary productivity [44]. On the other hand, Maerkang has a relatively high stocking density. A difference in fish farming density can have a significant impact on the microbial composition of the water environment. High-density farming usually leads to a decrease in microbial diversity, accumulation of pathogenic microorganisms, and an increase in organic matter-decomposing bacteria, while low-density farming helps to maintain a more balanced and diverse microbial community. Through a subsequent manual management process, the farming density of *H. bleekeri* in Maerkang can be appropriately reduced [45,46].

The present study employed metagenomic sequencing to analyze the microorganisms present in the water environments housing *H. bleekeri* in different regions. Microbial diversity plays a critical role in breeding success by improving health, boosting immunity, increasing reproductive capacity, and enhancing environmental adaptability. Ensuring microbial diversity in the environment is a key factor in successful breeding, particularly important in fields such as aquaculture. The application of metagenomic sequencing technology is used to elucidate the diversity and abundance of microorganisms in aquaculture environments in this study and may contribute to the artificial reproduction of *H. bleekeri*.

## 5. Conclusions

This study focused on the bacteriological profile in rearing water environments for *H. bleekeri*. Pseudomonadota was highly abundant in all the water samples. The common bacterial groups shared were found to be significantly more abundant in the aquaculture water in Jiguanshan, including Cyanobacteriota, Actinomycetota, Planctomycetota, Nitrospirota, and Verrucomicrobiota; the exception was Bacteroidota, which was more abundant in Maerkang. In addition, the Shannon diversity index and Simpson index of the microbial community in Maerkang were lower than those observed in Jiguanshan. It has been demonstrated that factors such as aquaculture density and water flow sources can influence the proportional microbial composition in the water environments. Understanding the microbiome diversity of aquaculture water will facilitate the advancement of knowledge on fish health and ecology, as well as more effective conservation of *H. bleekeri*.

## Data Availability

Metagenomic sequencing raw data have been deposited in the NCBI Sequence Read Archive (SRA) under Bio-Project Number PRJNA1165841 with BioSample accessions SAMN43934600–SAMN43934617.

## Supplementary Materials

The following supporting information can be downloaded at: https://www.mdpi.com/article/10.3390/genes15101314/s1, Table S1: Information in Maerkang and Jiguanshan; Table S2: Information on metagenomic sequencing; Table S3: Water microbial composition of the Maerkang and Jiguanshan fish farms at the phylum level; Table S4: Water microbial composition of the Maerkang and Jiguanshan fish farms at the Class level; Table S5: Water microbial composition of the Maerkang and Jiguanshan fish farm at the genus level; Table S6: Water microbial composition of the Maerkang and Jiguanshan fish farm at the species level; Table S7: Alpha diversity indices.
