# Supplementary material for: Metagenomic Sequencing Elucidated the Microbial Diversity of Rearing Water Environments for Sichuan Taimen (Hucho bleekeri)
Source: Genes (Basel). 2024 Oct 12;15(10):1314. doi: 10.3390/genes15101314 (PMC11507828; doi:10.3390/genes15101314)
Supplement: Supplementary file 1 [file genes-15-01314-s001.zip › genes-3215586-supplementary.pdf]

Supplementary Manuscript

Table S1. Information in Maerkang and Jiguanshan

	Maerkang	Jiguanshan
average daily temperature of April	9.9 ℃	18.5 ℃
water source	Class I tributary of the Chabao River	Class I tributary to the Anzi River
fish density	6-8 tails /m^3	0.3-0.5 tails /m^3
climate	subtropical highland climate	Humid subtropical climate
altitude	approximately 2500	approximately 1000
size of the farm	6667 m^2	6667 m^2

Table S2. Information on metagenomic sequencing

Sample ID	Raw_Base (G)	Clean_Base (G)	Clean_Q20 (%)	Clean_Q30 (%)	Clean_GC(%)	Effective (%)
M1	12.31	12.13	98.54	95.76	47.35	98.48
M2	12.91	12.62	98.31	95.01	46.29	97.76
M3	12.72	12.46	98.27	94.86	44.81	97.91
M4	12.67	12.45	98.44	95.36	46.08	98.28
M5	13.16	12.89	98.39	95.15	45.27	97.98
M6	12.12	11.97	98.73	96.28	43.56	98.78
M7	12.28	12.08	98.76	96.31	45.84	98.36
M8	12.48	12.26	98.54	95.64	44.15	98.22
M9	12.72	12.45	98.41	95.26	48.01	97.89
J1	13.14	12.87	98.28	95.18	51.21	97.91
J2	12.26	11.94	98.16	94.8	50.98	97.38
J3	12.22	11.91	98.15	94.77	51.75	97.47
J4	12.61	12.37	98.38	95.3	51.25	98.1
J5	13.06	12.66	98.01	94.29	54	96.88
J6	12.35	12.14	98.37	95.3	51.7	98.27
J7	12.18	11.97	98.38	95.34	51.81	98.28
J8	12.44	12.23	98.49	95.65	50.12	98.29
J9	12.3	12.05	98.29	95.06	52.63	97.95

Table S3. Water microbial composition of the Maerkang and Jiguanshan fish farms at the phylum level

Phylum level	Maerkang	Jiguanshan
Bacteroidota	0.413714	0.107759
Pseudomonadota	0.399961	0.361084
Cyanobacteriota	0.006727	0.105597
Actinomycetota	0.006507	0.044888
Planctomycetota	0.002982	0.01668
Nitrospirota	0.004144	0.022936
Uroviricota	0.008618	0.012177
Verrucomicrobiota	0.004616	0.01373
Bacillota	0.001649	0.010876
Fusobacteriota	0.001468	0.005533
Myxococcota	0.000685	0.004382
Chytridiomycota	0.001176	0.000794
Campylobacterota	0.001799	0.004799
Acidobacteriota	0.000435	0.002614
Nitrososphaerota	0.002408	0.003169
Mucoromycota	0.001244	0.001161
Bdellovibrionota	0.001661	0.002153
Ascomycota	0.000861	0.001038
Candidatus Melainabacteria	0.001321	0.000307
Zoopagomycota	0.000445	0.000181
Others	0.137579	0.278142

Table S4. Water microbial composition of the Maerkang and Jiguanshan fish farm at the Class level.

Class level	Maerkang	Jiguanshan
Flavobacteriia	0.376083	0.054721
Betaproteobacteria	0.190984	0.138795
Gammaproteobacteria	0.145018	0.115048
Cyanophyceae	0.006337	0.098607
Actinomycetes	0.005227	0.038008
Alphaproteobacteria	0.049628	0.094627
Nitrospiria	0.003654	0.02068
Cytophagia	0.006479	0.017254
Caudoviricetes	0.008618	0.012177
Chitinophagia	0.010591	0.004346
Fusobacteriia	0.001443	0.005444
Thermoleophilia	0.0002	0.002992
Planctomycetia	0.000403	0.003098
Sphingobacteriia	0.005574	0.004971
Bacilli	0.0007	0.005764
Opitutae	0.002341	0.006114
Acidimicrobiia	0.000248	0.001695
Bacteroidia	0.000855	0.00391
Myxococcia	0.000685	0.004382
Verrucomicrobiae	0.001071	0.00305
Others	0.183861	0.364317

Table S5. Water microbial composition of the Maerkang and Jiguanshan fish farm at the genus level.

Genus level	Maerkang	Jiguanshan
Flavobacterium	0.312083	0.037433
Aeromicrobium	0.000585	0.009755
Pseudomonas	0.020105	0.020199
Perlucidibaca	0.01926	0.00529
Limnohabitans	0.040858	0.006884
Methylotenera	0.019075	0.001448
Rhodoferax	0.030679	0.009645
Rheinheimera	0.009914	0.00434
Nocardioides	0.00127	0.012099
Brevundimonas	0.003654	0.011827
Crenothrix	0.023197	0.000811
Nitrospira	0.003404	0.019449
Pleurocapsa	0.001273	0.018299
Mycobacterium	0.000238	0.003154
Tychonema	0.000005	0.004905
Methylobacter	0.009541	0.000818
Limnobacter	0.000251	0.003359
Hydrocarboniphaga	0.000382	0.00265
Undibacterium	0.00672	0.003421
Paucibacter	0.003729	0.003994
Others	0.493777	0.82022

Table S6. Water microbial composition of the Maerkang and Jiguanshan fish farm at the species level.

Species level	Maerkang	Jiguanshan
Flavobacterium muglaense	0.046489	0.000272
Perlucidibaca aquatica	0.01854	0.005122
Oscillatoriales cyanobacterium	0.000017	0.013405
Pseudomonas sp. PGPPP3	0.009917	0.007257
Rhodoferax sp. PAMC 29310	0.021896	0.001851
Flavobacterium bernardetii	0.015421	0.001267
Crenothrix polyspora	0.02263	0.000773
Flavobacterium terrigena	0.012485	0.000464
Rheinheimera sp. 4Y26	0.005444	0.001908
Aeromicrobium erythreum	0.000211	0.003404
Methylotenera versatilis	0.00811	0.000304
Limnohabitans sp. Rim47	0.014342	0.001592
Nocardioides thalensis	0.000323	0.00272
Pleurocapsa sp. CCALA 161	0.001174	0.016047
uncultured Caudovirales phage	0.005612	0.005086
Flavobacterium degerlachei	0.010134	0.000088
Brevundimonas aveniformis	0.000181	0.001871
Flavobacterium aquatile	0.005015	0.000423
Nocardioides cavernaquae	0.000089	0.001839
Tychonema bourrellyi	0.000002	0.003296
Others	0.801968	0.931011

Table S7. Alpha diversity indices

sample	ACE	Chao1	Shannon	Simpson	Observed species	Goods coverage
M1	190.3805	190	1.457019	0.546295	189	1
M2	192.6745	193	1.670825	0.523253	192	1
M3	190.7927	190.5	1.467003	0.556208	190	1
M4	192	192	1.653738	0.575985	192	1
M5	196.7911	198	1.533156	0.556645	195	1
M6	190.0151	190	1.29547	0.50975	188	1
M7	197.1323	196.5	1.617595	0.564096	195	1
M8	193.7829	193.5	1.469824	0.536785	193	1
M9	194.2638	194	1.536789	0.55543	194	1
J1	195.4674	194.5	2.591824	0.692039	194	1
J2	196.25	196	2.588836	0.689279	196	1
J3	194	194	2.584852	0.683448	194	1
J4	193	193	2.423972	0.664637	193	1
J5	194	194	2.691943	0.757422	194	1
J6	191	191	2.452473	0.682396	191	1
J7	194	194	2.644532	0.724715	194	1
J8	193	193	2.529726	0.730463	193	1
J9	194	194	2.545949	0.681409	194	1
